# 促血小板生成药物临床应用管理中国专家共识（2023年版）

**DOI:** 10.3760/cma.j.issn.0253-2727.2023.07.002

**Published:** 2023-07

**Authors:** 中华医学会血液学分会血栓与止血学组

促血小板生成药物包括重组人血小板生成素（rhTPO）和血小板生成素受体激动剂（TPO-RA），其通过特异性结合血小板生成素（TPO）受体，调节巨核细胞增殖、分化与成熟，促进血小板生成。近年来，促血小板生成药物广泛用于治疗多种原因引起的血小板减少，可有效降低患者的出血风险、减少血小板输注、避免血液制品输注的不良反应。目前，全球已有5种促血小板生成药物获批进入临床应用，包括rhTPO、罗普司亭（Romiplostim）、艾曲泊帕（Eltrombopag）、海曲泊帕（Hetrombopag）、阿伐曲泊帕（Avatrombopag）及芦曲泊帕（Lusutrombopag）。深入了解此类药物的分子特征和机制特点，正确掌握用药指征，选择合适的剂量和疗程，规避不良事件，对临床医师十分重要。本共识由中华医学会血液学分会血栓与止血学组召集国内相关领域专家讨论制定，旨在规范、指导促血小板生成药物在国内的临床应用。本共识将重点介绍已在我国上市的促血小板生成药物（表1）。

**表1:** 目前中国获批上市的促血小板生成药物

药物	适应证	剂型	给药频率	给药时间	推荐剂量
rhTPO	CIT，ITP	注射剂	每日1次	无特殊要求	CIT 300 U/kg，ITP 300 U/kg
罗普司亭	ITP	注射剂	每周1次	无特殊要求	1～10 µg/kg
艾曲泊帕	ITP，SAA（IST疗效不佳）	片剂	每日1次	空腹	ITP 25～75 mg/次；SAA 75～150 mg/次
海曲泊帕	ITP，SAA（IST疗效不佳）	片剂	每日1次	空腹	ITP 2.5～7.5 mg/次；SAA 7.5～15 mg/次
阿伐曲泊帕	择期行诊断性操作或手术的CLDT	片剂	每日1次	与餐同服	40～60 mg/次

**注** rhTPO：重组人血小板生成素；ITP：原发免疫性血小板减少症；CIT：肿瘤化疗相关性血小板减少症；SAA：重型再生障碍性贫血；IST：免疫抑制治疗；CLDT：慢性肝病相关血小板减少症

一、药物种类及药理学特点

（一）rhTPO

rhTPO是利用基因重组技术由中国仓鼠卵巢细胞表达、经提纯制成的全长糖基化TPO，与天然TPO同源性达99％，分子量约为90 kD。rhTPO与TPO受体的胞外段结合后，引起TPO受体的构象改变，主要通过激活JAK/STAT、RAS/MAPK、PI3K/AKT三条下游信号通路，刺激多能造血干细胞、巨核祖细胞、多倍体巨核细胞的发育及成熟，促进血小板生成。rhTPO以300 U/kg皮下注射时，人体内消除半衰期为（40.2±9.4）h，故可每日或隔日给药1次。小鼠实验显示rhTPO主要以代谢物的形式经尿排泄，少量经粪排泄。

中国食品药品监督管理局（CFDA）分别于2005年及2014年批准该药用于治疗实体瘤化疗相关血小板减少及原发免疫性血小板减少症（ITP）。

（二）口服小分子非肽类TPO-RA

口服TPO-RA与人TPO受体的跨膜结构域相互作用，启动信号级联反应，诱导髓系祖细胞和巨核细胞的增殖和分化。TPO-RA不与内源性TPO分子竞争结合位点，与内源性TPO具有累加效应。口服TPO-RA具有种属特异性，只与人和黑猩猩的TPO受体跨膜区结合。与rhTPO及罗普司亭不同，口服TPO-RA对JAK、STAT磷酸化的激活较弱，对AKT通路没有刺激作用。

目前在我国获批上市的口服TPO-RA包括偶氮苯衍生物艾曲泊帕、海曲泊帕和噻唑衍生物阿伐曲泊帕。以上三种药物在人体内的药代动力学差异详见表2。

**表2:** 口服血小板生成素受体激动剂（TPO-RA）药代动力学特点

药物	分子量（D）	血浆消除半衰期（h）	生物转化/代谢	消除途径
艾曲泊帕	564	21.0～32.0	主要通过CYP1A2、CYP2C8、葡萄糖醛酸基转移酶A1、A3（UGT1A1、UGT1A3）代谢	粪便59％，尿液31％
海曲泊帕	580	11.9～40.1	主要通过多种葡萄糖醛酸基转移酶代谢	粪便89.05％，尿液8.62％
阿伐曲泊帕	765	19.0	主要通过CYP2C9和CYP3A4代谢	粪便88％，尿液6％

艾曲泊帕是全球第一个获批上市的口服TPO-RA，被美国食品和药物管理局（FDA）及欧洲药品管理局（EMA）批准治疗慢性ITP、丙型病毒性肝炎合并血小板减少、初诊及难治重型再生障碍性贫血（SAA）。在我国获批的适应证为≥6岁慢性ITP及既往经过免疫抑制治疗（IST）缓解不充分的成人SAA患者。

海曲泊帕是我国第一个自主研发的口服TPO-RA，其对艾曲泊帕的分子结构进行了修饰。于2021年6月获CFDA批准用于对糖皮质激素及免疫球蛋白等治疗反应不佳的慢性ITP、对IST反应不佳的成人SAA患者。

艾曲泊帕和海曲泊帕分子结构中均含有金属离子螯合基团，与抗酸药或含多价阳离子的其他产品（如奶制品和矿物质补充剂）合用时会显著降低药物暴露量。因此需空腹给药，给药前后与食物或其他药品至少间隔2 h以上。因其可增加体内储存铁的排出，对铁代谢正常的患者可引起铁缺乏（多见于儿童）[1]；而对铁过载的患者具有一定的祛铁作用[2]。有关艾曲泊帕的药代动力学研究显示，东亚裔、女性、老年（≥65岁）及肝功能损害患者的药物暴露量较高，肾功能损伤患者暴露量较低。

阿伐曲泊帕是新一代的口服TPO-RA，其分子结构中不含金属离子螯合基团，不受饮食限制，可与餐同服。年龄（18～86岁）、体重（39～135 kg）、性别、种族、肝功能损害及轻中度肾功能损害均不会对药代动力学产生有临床意义的影响。目前在我国获批用于择期行诊断性操作或手术的成人慢性肝病相关血小板减少症（CLDT）患者，在欧美国家还获批用于既往治疗反应不佳的成人慢性ITP患者。

体外研究显示，海曲泊帕及阿伐曲泊帕促进巨核细胞增殖分化的能力均强于艾曲泊帕，人胎肝CD34^+^细胞移植小鼠试验证实阿伐曲泊帕升高血小板计数的幅度高于艾曲泊帕[3]–[4]。但三者临床疗效的差异还需要头对头随机对照临床研究予以证实。

（三）小分子拟肽类TPO-RA

罗普司亭是利用重组DNA技术制成的Fc肽融合蛋白，含有两个相同的亚单位，每个亚单位分别由一个IgG1 Fc结构区和含有14个氨基酸的短肽（TPO模拟肽）构成，TPO模拟肽与TPO受体具有高亲和力并可使其激活。药物分子量59 kD，半衰期120～140 h，可每周1次皮下注射。罗普司亭是全球首个上市的TPO拟肽类药物，已在美国、欧盟、日本等多个国家地区获准上市，用于治疗脾切除及非脾切除的成人ITP、急性放射综合征、IST疗效不佳的SAA。CFDA于2022年批准罗普司亭及国内首仿药品用于治疗成人慢性ITP。

二、临床应用

（一）ITP

促血小板生成药物治疗ITP 1～2周起效，有效率达60％以上且疗效不受是否切脾影响。全球多中心随机对照临床研究显示，艾曲泊帕、罗普司亭可有效提高血小板计数、改善ITP患者的生活质量。此类药物停用后多不能维持疗效，需进行个体化维持治疗[5]。

1. rhTPO：300 U/kg每日1次，皮下注射，有效者行个体化维持。治疗14 d仍未起效的患者，应停药。rhTPO可单药或联合静脉注射免疫球蛋白（IVIg）、静脉甲泼尼龙用于重症ITP患者的紧急治疗[5]。

2. 艾曲泊帕：25～50 mg每日1次，空腹顿服。1～2周无效者加量至50 mg每日1次。最大剂量75 mg每日1次。如出血症状较重，可以50 mg/d起始，进行个体化药物调整，维持PLT≥50×10^9^/L。最大剂量应用2～4周无效者停药。

3. 海曲泊帕：2.5～5 mg每日1次，空腹顿服。最大剂量7.5 mg每日1次。应用2～4周无效者停药。

4. 阿伐曲泊帕：目前正在我国开展治疗慢性ITP的多中心Ⅲ期随机对照临床研究。起始剂量20 mg每日1次，与餐同服，2周无效者加量至40 mg/d，服用2～4周无效停药。

5. 罗普司亭：起始剂量为1 µg/kg每周1次皮下注射，每周增加1 µg/kg（最大剂量10 µg/kg），直到PLT≥50×10^9^/L。出血风险较高者，可从3 µg/kg起始，逐渐增加至5、7、10 µg/kg。

（二）再生障碍性贫血（AA）

TPO-RA通过与造血干/祖细胞表面的TPO受体结合，促进造血干细胞增殖分化及多能造血祖细胞扩增[6]，同时兼有免疫调节、诱导免疫耐受的作用[7]。另外，艾曲泊帕、海曲泊帕还具有铁螯合作用，可减少铁过载，有助于改善器官及骨髓功能[2],[8]。《再生障碍性贫血诊断与治疗中国指南（2022年版）》推荐无HLA相合同胞供者和年龄>40岁患者的首选治疗为TPO-RA联合IST[9]。

1. 艾曲泊帕：起始剂量为75 mg/d，根据疗效情况可每2周增加25 mg/d，最大剂量为150 mg/d。艾曲泊帕单药治疗IST无效的SAA，血液学缓解率为40％，获得缓解的中位时间为10周[10]。血小板恢复正常后缓慢减量，切忌骤然停药。

2. 海曲泊帕：起始剂量7.5 mg/d，每2周增加2.5 mg/d，最大剂量15 mg/d。单药治疗18周，血液学缓解率为43.6％，12个月无复发生存率为79.5％[11]。

3. rhTPO联合IST：治疗SAA时，15 000 U每周3次，皮下注射。治疗3、6、9个月的血液学缓解率分别为42.5％、62.5％、67.5％，同时伴有骨髓巨核细胞数量升高[12]。

4. 罗普司亭：起始剂量每周10 µg/kg，有效率为30％，用药9周可获得血小板反应[13]。

5. 阿伐曲泊帕：国内学者尝试阿伐曲泊帕联合IST治疗伴有肝功能损害的6例SAA患者，全部获得血液学反应伴肝功能好转[14]。

（三）CLDT

艾曲泊帕获FDA批准治疗丙型病毒性肝炎所致血小板减少。起始剂量为25 mg/d，每两周增加25 mg/d，最大剂量100 mg/d，在抗病毒治疗（长效干扰素联合利巴韦林）前应用，可显著提高丙型肝炎后肝硬化患者的起始血小板计数，有助于患者完成抗病毒治疗，提高病毒持久应答率[15]。但需警惕艾曲泊帕联合干扰素和利巴韦林可能增加丙型肝炎患者肝功能失代偿的风险。

全球多中心、随机、双盲、安慰剂对照、Ⅲ期临床研究（ADAPT-1、ADAPT-2）结果表明，阿伐曲泊帕显著降低成年CLDT患者围手术期血小板输注或因出血进行抢救的比例[16]。建议操作或手术前10～13 d开始口服。PLT<40×10^9^/L的患者，60 mg每日1次，连服5 d；PLT 40×10^9^/L～<50×10^9^/L的患者，40 mg每日1次，连服5 d。阿伐曲泊帕可能导致门静脉血栓形成，虽然风险低于其他TPO-RA，仍不建议慢性肝病患者长期口服阿伐曲泊帕维持血小板计数。

（四）肿瘤化疗相关性血小板减少症（CIT）

目前，只有rhTPO在我国获批CIT适应证。因缺乏足够的循证医学证据，TPO-RA在任何国家或地区均未获批CIT适应证。

1. rhTPO：恶性肿瘤患者化疗后血小板计数降至<75×10^9^/L时开始应用rhTPO 300 U/kg每日1次皮下注射，当PLT≥100×10^9^/L或较用药前升高50×10^9^/L时及时停药。如上一个化疗周期发生过2级以上血小板减少或出血风险较大，建议此次化疗结束后6～24 h应用rhTPO二级预防[17]。

2. 罗普司亭：Ⅱ期随机对照临床研究显示，罗普司亭可使93％的患者PLT在3周内升至≥100×10^9^/L[18]。美国国立综合癌症网络（NCCN）指南推荐罗普司亭用于治疗CIT，起始剂量2～4 µg/kg每周1次皮下注射，每周增加1～2 µg/kg，最大剂量10 µg/kg，直到PLT升至（100～150）×10^9^/L。但需警惕罗普司亭可增加实体瘤患者发生静脉血栓的风险[19]。

3. 阿伐曲泊帕：一项全球多中心、随机、安慰剂对照的Ⅲ期临床研究评估了阿伐曲泊帕治疗CIT的疗效与安全性。阿伐曲泊帕组与安慰剂组的主要研究终点达成和不良事件发生差异均无统计学意义[20]。我国开展的一项多中心、开放标签、单臂临床试验[21]中，阿伐曲泊帕治疗（60 mg/d，持续5～10 d）4周时累积有效率为70.3％，56.8％的患者PLT升至≥100×10^9^/L，恢复时间为（10.2 ± 6.4）d。

（五）骨髓增生异常综合征（MDS）

目前尚无一种TPO-RA获批MDS适应证。NCCN指南推荐艾曲泊帕、罗普司亭用于治疗伴有重度血小板减少的较低危（IPSS低危、中危-1）MDS。研究显示，艾曲泊帕（50～150 mg/d）及罗普司亭（250 µg起始，每周或每两周1次，最大剂量1 000～1 500 µg）可提升较低危MDS患者的血小板计数、降低出血风险、减少血小板输注[22]–[24]。

（六）造血干细胞移植后血小板重建不良

《造血干细胞移植后出血并发症管理中国专家共识（2021年版）》[25]推荐rhTPO及TPO-RA治疗造血干细胞移植后血小板重建不良。

1. rhTPO：300 U/kg每日1次皮下注射。多项研究证实，rhTPO有利于移植后血小板植入，可显著减少血小板输注、改善患者预后[26]–[27]。

2. 艾曲泊帕：50 mg/d空腹顿服，治疗无效者加量至75 mg/d，最大剂量为75～150 mg/d。连续应用2个月，无效者逐渐减停。国内一项Ⅱ期随机对照临床研究显示，与传统治疗相比，艾曲泊帕可显著提高造血干细胞移植后PLT升至≥50×10^9^/L的患者比例、减少血小板输注，但总生存（OS）率、无进展生存（PFS）率、复发率及无复发生存率没有显著改善[28]。

3. 阿伐曲泊帕：起始剂量20 mg/d，治疗1周无效者加量至40 mg/d，最大剂量60 mg/d。国内回顾性研究显示，总反应率（ORR）为68.9％，完全缓解（CR）率为39.3％，中位起效时间21 d[29]。

（七）妊娠合并ITP

国内小样本多中心前瞻性临床研究初步证实，对初始治疗无效的晚期妊娠合并ITP患者，给予rhTPO 300 U·kg^−1^·d^−1^×14 d，总体有效率达74.2％，无明显不良事件发生[5],[30]。

三、不良反应管理

（一）肝毒性

肝毒性是艾曲泊帕及海曲泊帕较常见的不良反应，主要表现为血清丙氨酸氨基转移酶（ALT）、天门冬氨酸氨基转移酶（AST）及间接胆红素升高。亚洲人种多见，大多为轻度，无临床症状，停药后可恢复。但对伴有肝脏基础疾病的人群，可能引起严重肝毒性和潜在致命性肝损伤。开始此类药物治疗前，应检测血清ALT、AST和胆红素，剂量调整期间上述指标每2周检测1次，达到稳定剂量后，每月检测1次。肝功能损害的患者慎用。

阿伐曲泊帕、rhTPO在临床研究中没有发生药物相关肝功能异常。药物代谢不受肝功能影响。

（二）白内障

艾曲泊帕及海曲泊帕治疗慢性ITP患者的临床研究均观察到受试者用药后新发白内障或白内障恶化，发生率分别为5％、3.6％。建议治疗前及治疗中定期进行白内障监测。

（三）血栓/栓塞

研究发现，TPO-RA可通过增加血小板微颗粒形成、提高血小板表面血小板糖蛋白Ⅵ（GPⅥ）及P选择素的表达，促进ITP患者血小板活化[31]。随着TPO-RA用药时间的延长，动、静脉血栓事件的发生率是未接受TPO-RA治疗患者的2～3倍。血栓发生与血小板计数及TPO-RA的剂量无关，30％～50％的ITP患者发生血栓/栓塞时血小板计数低于正常。血栓多发生于TPO-RA治疗后一年，有血栓/栓塞风险因素的患者为高发人群。因此TPO-RA治疗前，建议对患者进行血栓/栓塞的风险评估，个体化制定血小板升高的目标值及ITP相关治疗策略[32]。

（四）骨髓纤维化

10％～50％的ITP患者在应用TPO-RA治疗期间出现轻度骨髓网状纤维增生（MF-1级），少数（<10％）发展为MF-2级，罕见MF-3级或胶原纤维增生，骨髓纤维化与所应用TPO-RA的种类、剂量和疗程无关，绝大多数患者停用TPO-RA后骨髓纤维化可逆转[33]–[36]。目前不推荐ITP患者TPO-RA治疗期间对骨髓纤维化进行常规监测。在启动TPO-RA治疗前，如果骨髓活检显示>MF-2级或胶原纤维增生，不推荐应用TPO-RA治疗。TPO-RA治疗期间，如果出现血细胞减少或形态异常，建议及时行骨髓活检。如MF-3级或胶原纤维增生，需停用TPO-RA；如MF-2级，可以继续应用TPO-RA，但需在6个月内复查骨髓活检。

（五）恶性克隆增生

目前关于TPO-RA是否促进恶性克隆增生或克隆演化尚无定论。在应用TPO-RA治疗SAA及较低危MDS的临床研究中，没有观察到恶性克隆演化或向AML转化/进展风险的增加[37]–[38]。但对于高危MDS/AML，相关临床研究却得出相反的结果[39]–[40]。因此不推荐应用TPO-RA治疗伴血小板减少的中危-2或高危MDS及AML患者。在TPO-RA治疗SAA及较低危MDS期间，应定期检测骨髓细胞形态及细胞遗传学变化，必要时可行二代测序。

（六）血小板水平波动

相较于其他TPO-RA，ITP患者应用罗普司亭治疗期间更易出现血小板水平波动，主要与其给药间隔较长有关。血小板水平过度波动可导致出血/血栓风险增加，此时可更换为其他TPO-RA。

（七）中和性抗体生成

注射rhTPO或罗普司亭可能产生针对药物的一过性低滴度抗体，发生率为3％～10％。绝大多数仅为药物结合抗体，不影响药物疗效。极少数为药物中和抗体，导致血小板反应丢失。目前尚未发现药物抗体与内源性TPO存在交叉反应[41]–[44]。

四、剂量调整

遵循个体化原则，应用最小药物剂量维持患者PLT（50～150）×10^9^/L。

应用口服TPO-RA（海曲泊帕、艾曲泊帕、阿伐曲泊帕）治疗ITP时，如为低剂量爬坡（每日1片起始），减量时建议首先降低给药频次，逐渐延长给药间隔；如为高剂量起始（每日2～3片），减量时建议首先降低剂量（低剂量与原剂量交替应用），再减至低剂量持续应用，然后逐渐延长低剂量给药间隔。剂量调整期间，需密切监测血小板计数，尽量避免血小板过度升高或快速下降。

建议调整剂量后观察2周，评估剂量调整对血小板计数的影响，然后再考虑是否继续调整剂量。

治疗ITP时，若足剂量应用2周无效，应考虑停药或转换为另一种促血小板生成药物；治疗SAA、较低危MDS、CIT时；若足剂量应用2～3个月无效，考虑停药或转换为另一种促血小板生成药物。

此类药物停药后，血小板计数可能在一段时间内持续升高，为避免血栓风险，建议rhTPO治疗期间PLT≥100×10^9^/L时暂停给药，罗普司亭治疗期间PLT≥150×10^9^/L时暂停给药；口服TPO-RA在PLT≥250×10^9^/L时暂停给药。对于合并血栓风险的患者，可降低停药的血小板计数阈值[32]。停药后需密切监测血小板计数（每周至少2次），如血小板计数呈下降趋势，可酌情重启治疗。

五、疗效丢失的管理

TPO-RA治疗过程中发生疗效丢失的可能原因如下：①服药方式不正确，影响药物吸收：建议艾曲泊帕、海曲泊帕每晚睡前服用，服用前后2～4 h保证绝对空腹。不得将药物碾碎后混入食物或液体服用。不能用矿泉水送服药物。②合并感染：积极控制感染，注意个人防护，尽量减少或避免感冒、肠道感染等。③诱导产生C-MPL抗体或药物中和抗体：前者可导致rhTPO及TPO-RA的疗效下降或丢失[45]–[46]；后者见于应用rhTPO或罗普司亭治疗后。通过检测血浆中的相关抗体可以明确诊断。增加药物剂量/联合低剂量泼尼松（2.5～10 mg每日1次）或双醋瑞因（50 mg每日2次）可逆转药物反应[47]–[48]。如上述措施无效，需停用相关药物，待循环中的抗体代谢清除后重启治疗或尝试应用CD20单抗减少抗体生成。

六、TPO-RA相互转换

由于不同TPO-RA药物在作用靶点、体内代谢及升血小板效能方面存在差异，一种TPO-RA治疗无效或不耐受时更换或序贯其他TPO-RA治疗，可能使患者获益[5]。临床研究表明，因不同原因（疗效不佳或丢失、不良反应、给药方式等）进行TPO-RA转换治疗的ITP患者，仍能获得60％的血小板应答率[49]。

艾曲泊帕治疗无效的难治性SAA转换为罗普司亭每周20 µg/kg治疗3个月，70％的患者出现不同程度血液学反应[50]。小样本临床研究显示，IST联合艾曲泊帕/海曲泊帕治疗无效或不耐受的SAA患者转换为阿伐曲泊帕40 mg每日1次治疗，中位治疗6个月，70％的患者获得三系血液学反应，起效时间为3个月[51]。

七、妊娠、哺乳患者用药

动物试验证实TPO-RA具有生殖毒性且可经乳汁分泌，因此不建议用于妊娠人群，除非预期获益超过其对胎儿的潜在风险（仍需慎重）。服药期间及停药后两周内暂停哺乳。

八、ITP患者停药探索

马德里10年真实世界数据显示，29％接受TPO-RA治疗的ITP患者在长达28.4个月随访期内，可成功停药无复发[52]。英国关于TPO-RA减量及停药的共识推荐：接受TPO-RA治疗≥6个月、PLT稳定>50×10^9^/L、在过去6个月内无出血事件发生且无高危出血风险的患者可以尝试减停药物[53]–[54]。我国一项回顾性研究显示，停止其他ITP合并治疗后无复发、艾曲泊帕减量前PLT≥100×10^9^/L以及艾曲泊帕减量时剂量≤25 mg/d是获得无治疗缓解的预测因素[55]。随着ITP患者停用TPO-RA后持续缓解的证据增加，有望改变ITP长期治疗的传统模式，但如何筛选停药患者、明确停药标准、停药方案及停药后疗效预测因素、细化长期随访和管理策略仍需前瞻性大样本的随机对照研究予以证实。
